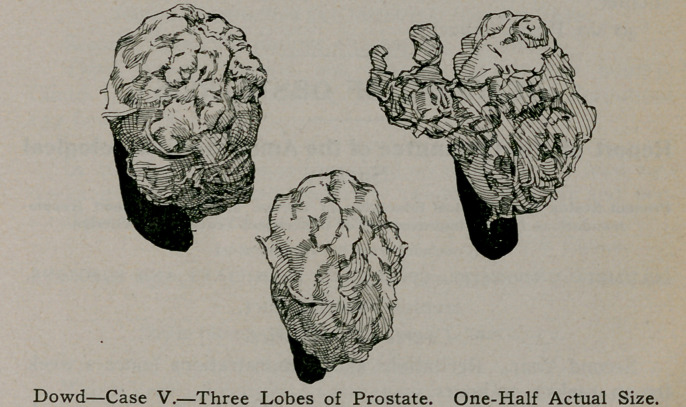# Presentation of Genitourinary Specimens1Read at the forty-second annual meeting of the Medical Association of Central New York, held at Auburn, October 19, 1909.

**Published:** 1910-07

**Authors:** J. Henry Dowd

**Affiliations:** Buffalo, N. Y.; Genitourinary Surgeon Sisters of Charity and Mercy Hospitals


### Presentation of Genitourinary Specimens[1]

By J. HENRY DOWD, M.D., Buffalo, N. Y.
Genitourinary Surgeon Sisters of Charity and Mercy Hospitals

IN presenting to the association these specimens, permit me to observe by way of prelude, that neither stone, nor foreign bodies, nor tumor of the bladder are new, or that this is the introduction of prostatectomy as a relief for hypertrophy, but to emphasize the fact that the bladder should be attacked from below more frequently than it is, thus hastening rapid closure of the wound, which is practically the initial method of removing bladder tumors; also the size of the stones and prostate which I show you are to be noted as unusual.

1. Read at the forty-second annual meeting of the Medical Association of Central New York, held at Auburn, October 19, 1909.

Case I.—S. S., aged 65, operation August, 1906. For the past fifteen years this patient had been unable to pass urine except by aid of the catheter, and for the past two years has had

**Dowd—Case I.—Tumor of the Bladder Shown in Situ.**

frequent attacks of hematuria. The condition was explained as marked hypertrophy of the prostate with tumor of the bladder, and operation advised. No operation was acceptable until acute abscess of the right lobe of the prostate made such necessary. The bladder was entered through the perineum and the tumor forcibly swept from its attachment by the index finger. Hemorrhage was quite marked, but entirely ceased in twenty-four hours, the patient being up and about with wound perfectly healed in twelve days. Although removal of tumor of the bladder by this method is practically new, the danger from hemorrhage is usually overcome by the rapidity with which the operation is performed and the promptitude with which wound closes. Up to date there has been no recurrence.

**Dowd—Case II.—Wax Taper. Exact Size.**

Case II.—J. A. G., age 19 years. For ten days this young man had very severe pains following urination which occurred every half hour or hour. Examination of the urine showed a large amount of pus, many blood corpuscles and crystals of phosphate of lime. All indications pointed to a foreign body in the bladder, yet the sudden onset precluded stone. The patient seemed ashamed of his condition, but finally, after much questioning admitted that he had passed a wax taper (eighteen inches long—specimen shown) into the urethra but which had slipped out of his grasp and disappeared. The bladder was entered by the perineal route and the taper removed. Recovery was uninterrupted, the wound being perfectly closed in ten days. The same remarks as to preference of route apply as in Case I.

**Dowd—Case III.—Bladder Stone. Exact Size.**

Case III.—Geo. C., age 4. For the past six or eight months this boy has cried every time he urinated which occurred on an average, every hour during the day and frequently at night. It was impossible to obtain further history on account of his inability to speak English, while the size of the organ prevented bladder examination. Examination of the urine showed crystals of phosphate of lime which, together with other findings, justified an opinion of stone in the bladder.

The viscus was entered by the perineal route, the left rudimentary prostatic lobe being divided. Uninterrupted recovery ensued, the boy being up with wound perfectly healed on the tenth day. The size of the stone shown (weight 90 grs.) occurring in practically a baby, the diagnosis from the urine and the route selected for operation, are points to be noted.

**Dowd—Case IV.—Bladder Stone. Exact Size.**

Case IV.—J. H., age 62. These five stones which are of the phosphatic variety weighing 1980 grains, were removed by the suprapubic route, a perineal tube being placed in the bladder for drainage. Recovery was uninterrupted, the patient in three weeks being able to hold his urine for four hours, the wound closing in four weeks.

It is not the result, nor the enormous size of these stones that I care to emphasise, but the prevention of a recurrence of such a condition. The patient's urine before operation was alkaline, having a very low phosphatic index. It is well known that alkalinity favors the precipitation of phosphatic crystals; that we rarely find them in acid urine, in the absence of phosphatic calculus of the kidneys or bladder. Acidity of urine is due mainly to acid sodium phosphate, therefore to change the urine from alkaline to acid is the answer. This is accomplished by raising the phosphatic index.

**Dowd—Case V.—Three Lobes of Prostate. One-Half Actual Size.**

Case V.—J. H. C., age 71 years. This specimen will almost speak for itself. (Normally the prostate weighs 6 drams, while the one I show weighs 26 drams). The three lobes were removed by the perineal route, the cavity being packed with iodoform gauze. A year and a half after the operation the patient sent this message: "I am as well as when a boy." I hold my urine for from 6 to 7 hours with no inconvenience."

Summing up the results of these cases I must offer the following remarks for consideration.

CONCLUSION.

1. Perineal wounds closing in twelve to fifteen days, suprapubic in from three weeks to eternity, must be convincing evidence that where it is possible to use the former route such should be employed.

2. The compressor muscle and posterior urethra being easily dilatable to at least 35 to 38 French scale, shows conclusively that much larger substances than were formerly supposed may be removed from the bladder by this route.

3. When a tube is placed in the bladder this viscus becomes almost completely collapsed. This, together with solutions such as adrenalin, if necessary, will prevent any serious hemorrhage.

4. Where tubes are necessary for drainage, either suprapubic or perineal, they should be removed as soon as possible, thus preventing the possibility of permanent fistula. In the first instance the sixth, and at the second, the third day may be considered a standing working rule.

5. The diagnosis of stone from the urine is not only possible in practically every case, but an opinion can be justly formed as to size—phosphatic aways large, uric acid, and oxalic acid smaller.

40 No. Pearl Street.

## Figures and Tables

**Figure f1:**
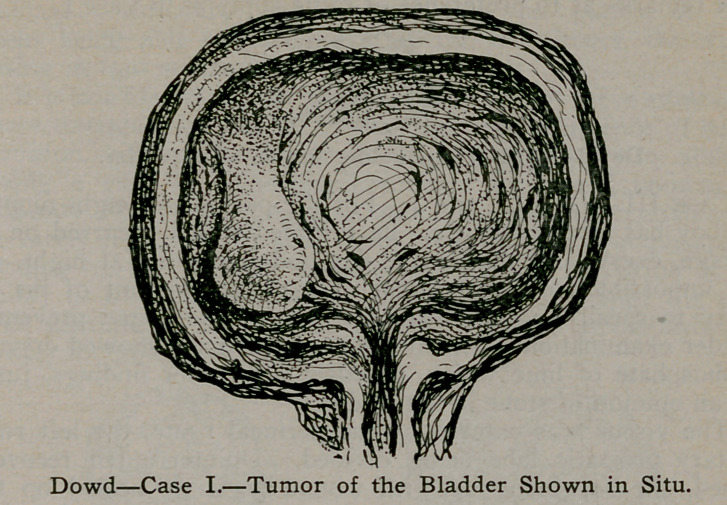


**Figure f2:**
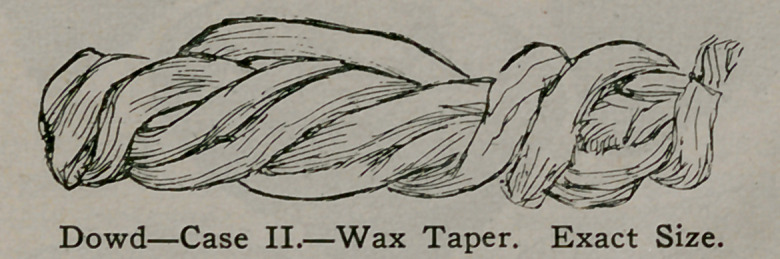


**Figure f3:**
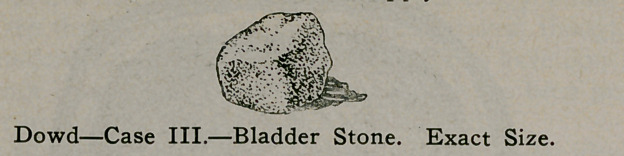


**Figure f4:**
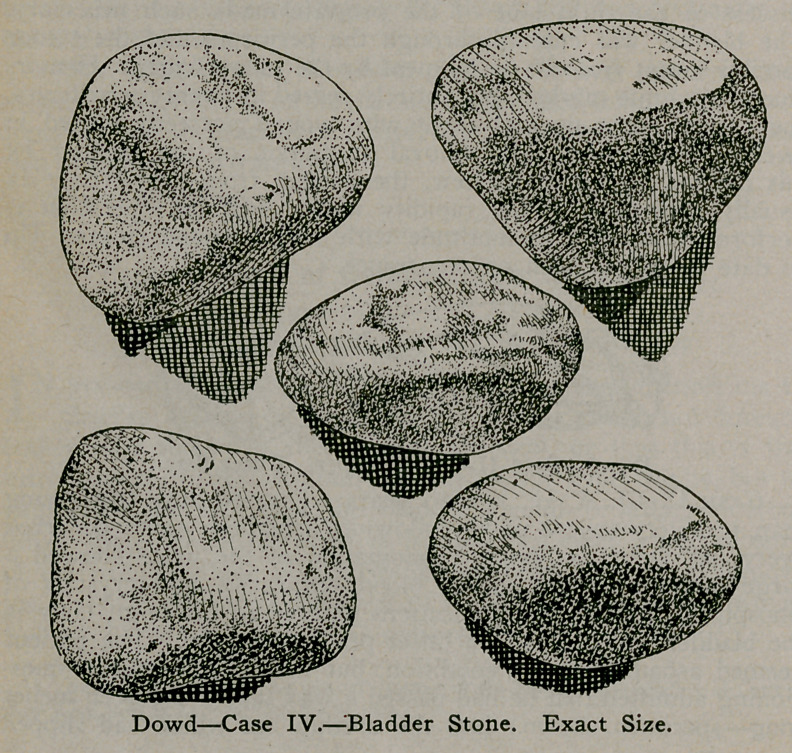


**Figure f5:**